# Tuberculosis Specific Interferon-Gamma Production in a Current Refugee Cohort in Western Europe

**DOI:** 10.3390/ijerph15061263

**Published:** 2018-06-14

**Authors:** Alexandra Jablonka, Christian Dopfer, Christine Happle, Georgios Sogkas, Diana Ernst, Faranaz Atschekzei, Stefanie Hirsch, Annabelle Schäll, Adan Jirmo, Philipp Solbach, Reinhold Ernst Schmidt, Georg M. N. Behrens, Martin Wetzke

**Affiliations:** 1 Department of Clinical Immunology and Rheumatology, Hannover Medical School, 30625 Hannover, Germany; sogkas.georgios@mh-hannover.de (G.S.); ernst.diana@mh-hannover.de (D.E.); atschekzei.faranaz@mh-hannover.de (F.A.); hirsch.stefanie@mh-hannover.de (S.H.); schmidt.reinhold.ernst@mh-hannover.de (R.E.S.); behrens.georg@mh-hannover.de (G.M.N.B.); 2 German Center for Infection Research (DZIF), Partner Site Hannover-Braunschweig, 30625 Hannover, Germany; solbach.philipp@mh-hannover.de (P.S.); wetzke.martin@mh-hannover.de (M.W.); 3 Department of Pediatrics, Neonatology and Allergology, Hannover Medical School, 30625 Hannover, Germany; dopfer.christian@mh-hannover.de (C.D.); happle.christine@mh-hannover.de (C.H.); jirmo.adan@mh-hannover.de (A.J.); 4 German Center for Lung Research, Partner Site Hannover BREATH, 30625 Hannover, Germany; 5 Hannover Medical School, 30625 Hannover, Germany; annabelle.schaell@stud.mh-hannover.de; 6 Department of Gastroenterology, Hepatology and Endocrinology, Hannover Medical School, 30625 Hannover, Germany

**Keywords:** tuberculosis, LTBI, refugee, asylum, infection, IGRA, infectious diseases, migrant

## Abstract

Background: In 2015, a high number of refugees with largely unknown health statuses immigrated to Western Europe. To improve caretaking strategies, we assessed the prevalence of latent tuberculosis infection (LTBI) in a refugee cohort. Methods: Interferon-Gamma release assays (IGRA, Quantiferon) were performed in *n* = 232 inhabitants of four German refugee centers in the summer of 2015. Results: Most refugees were young, male adults. Overall, IGRA testing was positive in 17.9% (95% CI = 13.2–23.5%) of subjects. Positivity rates increased with age (0% <18 years versus 46.2% >50 years). Age was the only factor significantly associated with a positive IGRA in multiple regression analysis including gender, C reactive protein, hemoglobin, leukocyte, and thrombocyte count and lymphocyte, monocyte, neutrophil, basophil, and eosinophil fraction. For one year change in age, the odds are expected to be 1.06 times larger, holding all other variables constant (*p* = 0.015). Conclusion: Observed LTBI frequencies are lower than previously reported in similar refugee cohorts. However, as elderly people are at higher risk for developing active tuberculosis, the observed high rate of LTBI in senior refugees emphasizes the need for new policies on the detection and treatment regimens in this group.

## 1. Introduction

Currently, Western Europe experiences immigration of a large number of immigrants from economically less developed or war-stricken countries such as Syria, Afghanistan, and Iraq [1,2]. In their countries of origin and during their escape or emigration, many migrants lacked access to regular health care and routine vaccination services [3]. During migration, malnutrition, overcrowding, physical and psychological stress, poor water supply, and poor sanitation predispose refugees to infectious diseases [4]. Especially tuberculosis (TB), an infectious disease occurring extremely rarely in immunocompetent persons in Western countries, represents an increasing problem in refugee health care [5]. Based on the recent increase in refugee TB cases, the awareness for this disease grows in Western Europe. In this regard, the identification of migrants at particular risk for TB and their further diagnostic, evaluation, and treatment is a matter of interest [6,7]. Data on the frequency of the TB status of migrants entering Europe is scarce and consequently, diagnostic regimens in migrants have not been harmonized.

In our current work, we performed tuberculosis specific interferon-gamma release assays (IGRA, Quantiferon) to assess the latent or active TB infection in a large, unselective subset of refugees representative of the current refugee crisis in Western Europe. The presentation of this data may help to assess the general risk of TB infections in migrants currently entering Western Europe and, more importantly, may on the long run support evidence-based harmonization of migrant screening for TB.

## 2. Material and Methods

### 2.1. Participants

#### 2.1.1. Study Population and Sample Collection

A total of *n* = 232 refugees underwent routine interferon-gamma release assay (IGRA) testing in four Northern German reception centers in August 2015. All subjects presented with acute complaints, mainly common colds or skin diseases such as scabies and were offered a routine blood checkup including the complete blood cell count, C reactive protein, and an IGRA. Further routine testing included the serological analysis of antibodies against vaccine-preventable diseases and parameters indicative for hepatitis. After *n* = 232 subjects, the local health authorities recommended stopping the analysis due to the limited consequence of LTBI in this setting and recommended X-ray examination only.

#### 2.1.2. Data Collection

The interferon-gamma Release Assay QuantiFERON-TB Gold Elisa was used (Quiagen, Hilden, Germany) and a whole blood interferon-gamma test measuring responses to ESAT-6, CFP-10, and TB7.7 (p4) peptide antigens. The laboratory had been certified for routine testing according to DIN EN ISO 15189:2014. For test interpretation, the QFT Analysis Software was used as recommended by the manufacturer, and the age and gender dependent normal values were classified according to the manufacturer suggestions. Specificity was estimated by the manufacturer to be >98%, the sensitivity for active TB was >80%, and the Quantiferon positivity can aid in diagnosing latent or active TB. Complete blood cell counts were obtained by automated analysis and confirmed by microscopic differential blood counts, if necessary. High sensitive CRP was determined by latex-enhanced immunoturbidimetry. All data were extracted from electronic routine patient records. For personal data protection, all data were anonymized before analysis. The date of birth and gender were kept available for analysis. In *n* = 5 patients, information on gender and in *n* = 18 patients, the data on age were unavailable or inconsistent in the records.

### 2.2. Analysis

Statistical analyses were processed using SPSS version 23.0 or GraphPad Prism version 5.02, the graphs were created using Microsoft Excel version 2003 and/or GraphPad Prism version 5.02. Calculation of LTBI prevalence was conducted by descriptive statistics. Ninety-five percent confidence intervals and standard errors were estimated by bootstrapping (simple, 1000 computations). Fisher exact testing was used for nominal variables for comparison between groups. Multiple regression (backwards, Wald, exclusion <0.10) was used for comparison between groups. Metric values were compared by Student’s *T*-testing. *P* values < 0.05 were considered significant.

### 2.3. Study Approval

The Institutional Review Board (Ethics Committee) of Hannover Medical School approved this analysis (#2972-2015). All patient information was anonymized prior to analysis.

## 3. Results

**Figure 1:**
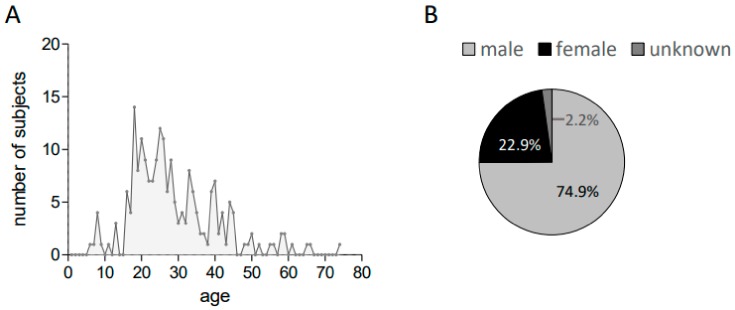
(**A**) The age and (**B**) gender distribution within the analyzed cohort.

IGRA testing was performed in *n* = 232 refugees. Subjects had a median age of 26 years (range 6–74 years, IQR 20–34.75). A total of 74.9% of the tested migrants were male. Figure 1 depicts the age and gender distribution within the cohort.

**Figure 2:**
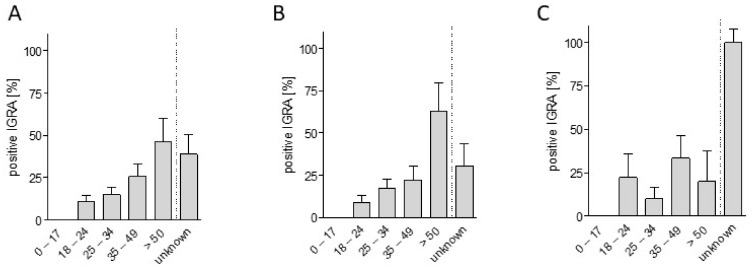
(**A**) Rate of positive IGRA test results in age- specific subgroups of the analyzed refugee cohort; (**B**) Age-related IGRA positivity in male and (**C**) female migrants (bars display the mean plus standard error).

In *n* = 223 (96.1%) subjects, valid IGRA results were obtained. All patients showed in vitro cellular reactivity (reaction to positive control “Mitogen” >0.5 IU/mL), and all negative controls showed no significant reaction. No active, smear-positive tuberculosis was detected in the group. Two refugees were HIV positive, but had a negative IGRA result. Overall, IGRA testing was positive in 17.9% (95% CI = 13.2–23.5%) of subjects. There were no significant differences of the prevalence in male and female refugees [16.8% (95% CI = 11.2–22.8) versus 19.6 (95% CI = 9.8–30.8)]. As shown in Figure 2A, the proportion of positive IGRA testing increased with age (Fisher exact, *p* = 0.001) (Table 1). While no refugees <18 years displayed positive IGRA testing, 46.2% of refugees >50 years had a positive result. This clear effect was also observed in the male subset of tested refugees that displayed an age-related increase of IGRA positivity with 0% and 9.1% in minor aged and young adults aged 18–24 years as compared to 62.5% in the oldest age group (Figure 2B). In women, however, this effect was not clear, most probably due to the low number of the total subjects (*n* = 10, Figure 2C).

**Figure 3:**
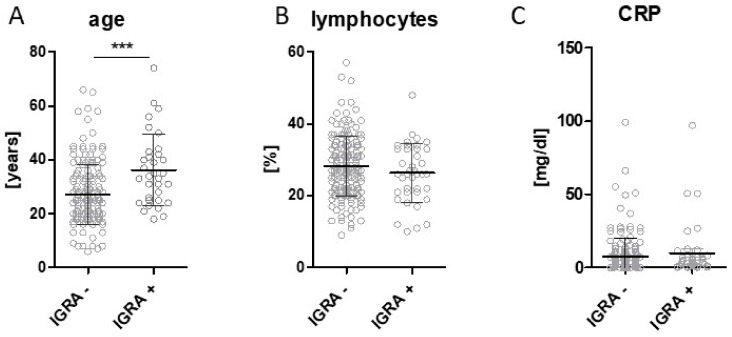
The analysis of subject-specific parameters in refugees with negative (IGRA −) or positive (IGRA +) testing results. (**A**) age; (**B**) CRP; (**C**) hemoglobin (bars display the mean plus standard deviation, Hb: hemoglobin, *** *P* < 0.001).

When comparing the subject-specific parameters in IGRA positive versus IGRA negative subjects, only age yielded a highly significant difference in statistical testing. (Figure 3).

Age remained the only factor significantly associated with a positive IGRA in multiple regression analysis including gender, C reactive protein, hemoglobin, leukocyte, and thrombocyte count and lymphocyte, monocyte, neutrophil, basophil, and eosinophil fraction. For one year change in age, the odds are expected to be 1.06 times larger, holding all other variables constant (*p* = 0.015).

## 4. Discussion

We present data on the age and gender dependent prevalence of TB specific IGRA positivity in migrants entering Europe during the current refugee crisis. The majority of refugees in our cohort were young adults and in all age groups, most migrants were male. As such, the analyzed subset is representative of the current population of refugees seeking asylum in Western Europe which, to the vast majority, consists of young men [8,9,10]. Overall, we observed 17.9% of positive IGRA tests in the analyzed cohort with an age-dependent increase of positive test results in elder refugees. This is similar to previous publications that report on IGRA positivity rates of 20% in migrants entering the Netherlands in 2012 [11].

In refugees, communicable diseases are a particular threat [3,12,13,14,15] as they often times have limited access to health care services or appropriate nutrition or sanitation [16]. While TB is rare in Western countries, the global burden of latent tuberculosis infection has been estimated to be as high as 25% [17]. In 2016, 6.3 million new TB cases were reported [18]. To tackle this worldwide problem, the World Health Organization’s (WHO) has formulated updated guidelines and an “End TB Strategy” [19,20]. To reach the WHO target of pre-elimination by 2035, the identification and treatment of active or latent TB infection in immigrants is central [21]. In Western countries, the vast majority of TB cases occur in migrants, for example nearly 65% of active TB cases in the US in 2013 and 73% of all TB infections in the Netherlands in 2009 were in foreign-born persons [22,23].

A central mechanism of migrant health, as well as the TB prevention in the receiving population, is the screening of immigrants for latent and active TB [22,24]. During the current extent of migration to Western Europe, receiving countries have set up different regimens to assess the health status of newly arriving refugees with or without implementation of routine testing for TB infection. Screening regimens are not harmonized. As recently assessed by the WHO and European Respiratory Society (ERS), 86.1% of 38 European national TB screening representatives reported screening for active TB, 50% screened for latent TB, and only 22.2% reported outcomes of latent TB treatment [25]. In 61.1% of European countries taking part in the WHO/ERS survey, screening for active or latent TB was performed in refugee centers. While 75% of countries answered that screening for TB was performed according to national and international guidelines, only 52.7% gave the same answer with regard to latent TB diagnostics [25]. In Germany, asylum law and national infection prevention plans state that all immigrants (except pregnant women) aged 16 years or older living in shared accommodation facilities such as reception centers or shelters for asylum seekers must undergo mandatory chest radiographs, primarily to identify active pulmonary tuberculosis (German Asylum law, § 62 Abs. 1 AsylG). Further measures of upon-entry screening for TB, especially in children or pregnant women, are governed by different policies at the level of the 16 federal states.

The usefulness of screening newly arriving immigrants for latent TB by IGRA is a matter of debate. Mulder et al. estimated the number needed to be treated to prevent one case of TB within 2 years in migrants in the Netherlands, given a positive IGRA and an efficacy of 60% of prophylactic treatment, to be around 350 [11]. Follow up evaluations may include chest radiographs and induced sputum analysis. The assay used in our current work has been described to have a specificity for active TB of around 99% with a sensitivity of about 80% [26,27]. A positive IGRA at entry was associated with an up to 26-fold increased risk for active TB for refugees in the Netherlands in 2012 [11]. In contrast to skin testing (Mantoux tuberculin sensitivity testing), the IGRA assay is not affected by TB vaccination. However, IGRA testing cannot discriminate between TB positive individuals that were recently infected and those with long-standing TB. This is a clinically important distinction as recently infected patients carry a higher risk of reactivation disease progression [28]. As such, appropriate follow up diagnostics and treatment regimens need to be implemented to further evaluate the TB status of IGRA positive refugees.

Unfortunately, we were unable to follow up the refugees with positive IGRA screening in our cohort and cannot report on the chest radiograph or microbiological results or clinical data of these subjects. The observation of zero positive IGRA results in children and adolescents in our cohort is pleasant, previous publications reported latent TB rates of 2.7–6.8% and 0.5% of TB in refugee children entering Europe during the current crisis [29,30]. However, the high IGRA positivity rates in elder refugees of our cohort are critical. Overall, we observed an age-related increase in IGRA positivity with 46.2% of senior refugees above the age of 50 years (62.5% of males >50 years). This finding is of particular concern, as older persons are at increased risk for TB reactivation [31,32]. TB reactivation in elder refugees does not only pose a risk for immigrant health but also for the receiving population, and our data support the notion that health caregivers, during the current refugee crisis, should be particularly aware of the risk for TB in older refugees. However, our analyses are limited by the fact that we only present data on a comparably small number of subjects, only *n* = 10 female refugees (*n* = 10) were IGRA positive, and only *n* = 21 underaged refugees were tested. Another limitation of our work is the fact that we were unable to link the IGRA results to subject-specific countries of origin. It has been reported that migrant TB rates reflect those of their countries of origin [11,21,33,34,35]. For example, refugees from Sub-Saharan Africa have been reported to be at particular risk for latent TB infections [30,36]. Future studies should include information on the home countries of immigrants to facilitate IGRA based TB risk estimation in specific refugee subgroups.

## 5. Conclusions

Taken together, we here present the current data on the rates of TB specific IGRA positivity in a representative refugee cohort in Western Europe. The growing population of refugees currently entering Europe challenges receiving healthcare systems and requires effective medical programs based on reliable epidemiological data [16]. Only by stringent screening with appropriate follow-up evaluation and effective therapy, TB associated morbidity can be reduced in the immigrating and receiving population [24]. We hope that our dataset supports the adaptation of appropriate screening and caretaking regimens.

## Figures and Tables

**Table 1:** Rate of positive IGRA test results in age-specific subgroups of the analyzed refugee cohort.

Age	Total *n*	% Positive IGRA
0–17 years	21	0
18–24 years	65	10.8
25–34 years	67	14.9
35–49 years	39	25.6
≥50 years	13	46.2
unknown	18	38.9
